# Correction for Ligios et al., “Sheep with Scrapie and Mastitis Transmit Infectious Prions through the Milk”

**DOI:** 10.1128/jvi.00731-25

**Published:** 2025-05-28

**Authors:** Ciriaco Ligios, Maria Giovanna Cancedda, Antonello Carta, Cinzia Santucciu, Caterina Maestrale, Francesca Demontis, Mariangela Saba, Cristiana Patta, James C. DeMartini, Adriano Aguzzi, Christina J. Sigurdson

## AUTHOR CORRECTION

Volume 85, no. 2, p. 1136-1139, 2011, https://doi.org/10.1128/jvi.02022-10. Page 1138, Fig. 2: Since publication, we discovered irregularities in the Western blot images in Fig. 2B (“Scrapie” group). The blots contained samples from a comparison sheep group that had ingested milk from mastitis-free, scrapie-infected sheep and were blotted in the Ligios lab. Due to the age of the study, the original sheep samples are no longer available; thus, it is not possible to repeat the experiment. Replicate Western blot experiments were performed at the time of the original study, which upheld the conclusion that there was no PrP^Sc^ in sheep ingesting milk from mastitis-free sheep. The conclusion that there was no PrP^Sc^ in the brain or tonsil of this sheep group is also supported by the negative IHC shown in panel A. The conclusions of the paper are not impacted; sheep with lentiviral mastitis and scrapie secrete prions into milk to infect suckling lambs.

